# Do preschool teachers in Southwest China need more mental health education? An online cross-sectional survey 1 year after the COVID-19 pandemic

**DOI:** 10.3389/fpsyg.2022.907838

**Published:** 2022-08-05

**Authors:** Yao Yu, Tingting Wu, Jing Gao, Shanshan Wang, Yang Zhou, Jiajun Zhang

**Affiliations:** ^1^Children and Adolescent Physical Health Research Center, Chongqing University of Education, Chongqing, China; ^2^Faculty of Education, Southwest University, Chongqing, China; ^3^Department of Food and Nutrition, College of Medical and Life Sciences, Silla University, Busan, South Korea; ^4^Collaborative Innovation Center for Child Nutrition and Health Development, Chongqing University of Education, Chongqing, China; ^5^Children’s Hospital of Chongqing Medical University, Chongqing, China; ^6^School of Public Health and Management, Chongqing Medical University, Chongqing, China

**Keywords:** preschool teachers, mental health education, psychological problems, overtime, Southwest China

## Abstract

This study intended to explore the current status of psychological problems of preschool teachers in Southwest China 1 year after the COVID-19 pandemic and to assess the association between mental health education and psychological problems and symptoms of psychopathology. A total of 614 preschool teachers from Southwest China were enrolled to complete the questionnaires of the Chinese Symptom Checklist (SCL-90). Notably, 60% of the respondents reported psychological distress with GSI T-scores ≥ 63, especially the high score was reported on obsessive-compulsive disorder, interpersonal sensitivity, and phobic anxiety. Although less than half of the respondents have received mental health education last year, the teachers who received the mental health education reported lower GSI *T*-scores(β = −1.303, 95% CI: −2.208, −0.397). The results demonstrated the significance of constructing the education of promoting mental health of preschool teachers, and enlightening government or managers of kindergartens to relieve the psychological problems of preschool teachers through mental health education, especially for those with the pressure of overtime. It is recommended that local governments or kindergartens should organize more mental health education for kindergarten teachers to improve their mental health as well as their teaching professionalism.

## Introduction

Teaching is a stressful and challenging profession. The mental health of teachers is an essential factor affecting the quality of education. The mental health of preschool teachers plays an important role in their educational practice and professional development in early childhood education. Teachers’ wellbeing shapes children’s development by influencing a teacher’s ability to create and maintain high-quality early care and education environments (Rhodes and Huston, 2012; Harding et al., 2019). And it has been pointed out that social-emotional competence and wellbeing of teachers are necessary to facilitate positive interactions with the children. The mental health status of preschool teachers can affect the quality of care for young children (Lara et al., 2017; Laybourn et al., 2019).

However, many preschool teachers worldwide undergo mental disorders due to the demanding but depreciated property of the preschool teaching profession (Chou et al., 2016). Previous studies pointed out that the prevalence of mental disorder among preschool teachers is 53.2% in China (Li et al., 2020), 60.0% in Japan (Yaginuma-Sakurai et al., 2020), and 50% in Italy (Converso et al., 2015), respectively. Teachers’ mental health was significantly associated with effort-reward imbalance (Yaginuma-Sakurai et al., 2020), social support, economic status (Peele and Wolf, 2020), and individual mindsets (Kim et al., 2020). Even worse, in the beliefs of preschool teachers, they place less emphasis on psychosocial health (Chan and Kitzmann, 2010). A previous study showed that preschool teachers often face undue pressure from parents due to unreasonable expectations of parents from the preschool teachers and taking inappropriate pedagogical actions against them (Hsu et al., 2019).

The COVID-19 pandemic and lockdown, childcare, and school closures have affected the lives of children and their families around the world, which has brought about a sense of fear and anxiety around the world. These phenomena might lead to short-term as well as long-term psychosocial and mental health implications for children (Singh et al., 2020; Vasileva et al., 2021). As primary caregivers and educators in addition to parents, teachers have to adapt to many other changes that could potentially make them more vulnerable to psychological distress during the COVID-19 pandemic time. Studies have demonstrated that Chinese teachers reported high psychological distress, especially in anxiety, sleep disturbance, and somatic symptoms during the COVID-19 outbreak (Lizhi et al., 2021; Wei et al., 2021).

To cope with a possible mental health crisis that may arise during the COVID-19 epidemic, the government of China has responded quickly and comprehensively, and appropriate mental health policies were released for children and teachers (Mo, 2020; Qiu et al., 2020). The government of China emphasizes providing teachers and students with psychological assistance and counseling, which was issued on May 7 in 2020, MINISTRY OF EDUCATION in THE PEOPLE’S REPUBLIC OF CHINA in conjunction with the National Health Commission for *COVID-19 Prevention and Control in Primary and Secondary Schools and Kindergartens and Child Care Institutions* (Ministry of Education of the People’s Republic of China, 2020). This plan encourages local governments, primary and secondary schools, and childcare institutions to carry out various forms of health education practices to popularize the prevention and control of COVID-19, especially mental health education, to stabilize the minds and emotions of children and teachers. However, research is still limited on mental health status and the effect of mental health education by the institutions among teachers, especially among preschool teachers.

Education is one of the main pillars of sustainable development in a knowledge-based economy. Mental health education has been demonstrated as one of the main pillars of sustainable development in mental health improvement and helps teachers to evaluate their occupations properly. In addition, a previous meta-analysis found that preschool teachers in Southwest China were less researched than in other regions in China (Fan et al., 2016). Thus, this study focused on preschool teachers in southwest China, to evaluate the psychological problems and assess the association between mental health education and the SCL-90 scores. We hope that this study will add valuable information to help develop future interventions that help restore and maintain educators’ wellbeing and mental health.

## Materials and methods

### Study population and procedure

A web-based survey was carried out, by convenience sampling, to set the notifications on WeChat, which is a social media platform, in December 2020. The study population included the preschool teachers who had agreed to participate in online surveys voluntarily. Based on the single population proportion formula (Daniel, 1999), n=(Z⁢α2⁢ 2M⁢O⁢E2*p⁢(1-p)). We set the α = 0.05, $Z\,\frac{\alpha }{2}$= 1.96, and MOE = 0.05, *p* = 50%, the calculated *n* = 383. The study provided an anonymous and detailed description of the purpose of the research and the tips about filling the questionnaire were shown on the questionnaire completion website. Volunteer preschool teachers fill out the online questionnaire that was designed and later diffused by a web link to different social media. A total of 700 questionnaires were collected in this survey, and a total of 648 questionnaires were recovered, with a response rate of 92.57%. The exclusion criteria for this survey data are as follows: (1) the questionnaire data with logical errors; (2) the questionnaire data with inconsistency on the reconfirmation question such as gender and age; (3) participants whose frequency of watching a movie is less than once a year and more than three movies in a day. A total of 614 questionnaire samples were included in this research (46% from Chongqing, 36% from Sichuan, and 18% from Guizhou; the response rate for the valid sample was 94.75%). This study was approved by the Ethics Committee Review Committee (202012HS02). All the participants signed the informed consent forms before filling the formal survey online. And a pilot survey was conducted including 30 samples 2 months before the formal survey.

### Measures

#### Mental health education

Mental health education refers to further enhancing the professional competence of early childhood teachers in China, and local governments encourage early childhood teachers to participate in mental health-related continuing education. These continuing education activities are usually organized by the local governments and educational institutions, which are based on COVID-19 Prevention and Control in Primary and Secondary Schools and Kindergartens and Child Care Institutions (Ministry of Education of the People’s Republic of China, 2020) to stabilize the minds and emotions of children and teachers. In this study, early childhood teachers were asked to answer, “Have you received mental health-related education organized by in your institution or units and higher-level authorities in the past year?” (Yes or no).

#### Individual characteristics

The individual characteristics data gathered *via* a questionnaire comprised sociodemographic variables and lifestyle. Sociodemographic variables include gender (dummy coded as 1 = male, 2 = female), age (in years), marital status (dummy coded as 1 = unmarried, 2 = married, and 3 = separated, divorced, or widowed), and the number of children (dummy coded as 1 = none, 2 = one child, and 3 = two or more children). Lifestyle included the hours of sleeping, the frequency of moderate exercise (dummy coded as 1 = none, 2 = once or twice a week, 3 = three to four times a week, and 4 = more than five times a week), and the frequency of eating breakfast (dummy coded as 1 = everyday, 2 = 5–6 times/week, 3 = 3–4 times/week, 4 = 1–2 times/week, 5 = never eating).

#### Working background variables

The working background characteristics of teachers were measured by teachers’ responses about their educational attainment, the attribute of kindergarten, the number of years they had worked in the field of early childhood education, work hours of a workday, and their monthly salary. Teachers’ educational attainment was dummy coded into two variables, namely, (a) medium-level education (compared with teachers with a degree) and (b) high-level education (had received a college or graduate degree). The kindergarten attribute was categorized into government-run and private-run kindergartens. Monthly salary was categorized into four levels: (a) < 3,000 yuan, (b) 3,000–4,500 yuan, (c) 4,501–6,000 yuan, and (d) > 6,000 yuan.

#### Mental health symptom checklist (SCL-90)

The Symptom Checklist SCL-90, a 90-item self-report symptom inventory, was used to evaluate the mental health of teachers, which has been translated into Chinese and provides an empirical test of validity (Yuanyuan et al., 2018). A total of nine primary symptom dimensions were included in the scale. The nine symptom dimensions are somatization, obsessive-compulsive disorder, interpersonal sensitivity, depression, anxiety, hostility, horror, paranoia, and psychosis. And seven additional items assess disturbances in appetite and sleep. The sum of all nine subscales is the Global Severity Index (GSI), which can be used as a summary of the test, reflecting overall psychological symptoms. As reported in previous studies, the raw GSI scores were also converted into T-scores (mean = 50, SD = 10) (Monteiro et al., 2017; Tian et al., 2020). Participants with GSI T-scores ≥ 63 were defined as having clinically significant psychological distress (high distress); the cut-off point has been proven as a valid and reliable marker for identifying severe psychological condition that requires assessment and possible intervention, and it has been used extensively in psychological studies in recent years (Assari, 2018; Tian et al., 2020). Besides, the mean score of subscales over two points indicates “mild symptoms,” and more than three points indicates “at least moderate symptoms.” In this study, α coefficients of the total scale and each factor were 0.99, 0.92, 0.89, 0.90, 0.92, 0.91, 0.87, 0.88, 0.86, and 0.90, respectively.

#### Data analysis

All the data were entered two times using Microsoft Office Excel 2016 (Microsoft, Redmond, WA, United States). All statistical data were analyzed using two-sided t-tests in the Stata statistical software (Stata, version 15.1, Cary, NC, United States). Descriptive statistics were applied to depict characteristics of sociodemographic variables and distributions of the scale scores with measures of mean, standard deviation, frequency, and percentage. Continuous variables were compared using ANOVA. The variability of the categorical variables was tested using the chi-square test. An independent-sample t-test was used to compare the mean scores and comparison with Chinese normative data. The stepwise regression model was used to analyze the factors that affect the score of SCL-90. Previous studies showed that sociodemographic characteristics and health-related arrangements and kindergarten regulations may have different effects on the mental health (Jeon et al., 2018). The following set of models was used: (i) Model 1 was adjusted for sociodemographic characteristics, which include age, gender, number of children, marital status, and BMI; (ii) Model 2 was further adjusted for education level, working years, monthly income, kindergarten regulations, and Model 1 variables; (iii) Model 3 was further adjusted for all variables in Model 2 and lifestyle. The results are presented as correlation coefficients (β) and 95% CI. Statistical significance was set at *p* < 0.05.

## Results

A total of 614 preschool teachers, including 64 men and 550 women, were included in our study. Only half of the participants had received mental health education. The mean age of teachers was 26.98 years (SD = 7.66). Most (93.16%) of the respondents have a high-level education. The majority of them (62.87%) were unmarried. A small majority of teachers came from private-rolled kindergartens (56.51%). The monthly income of most of the teachers (78.99%) was less than 4,500 yuan. Notably, 69.38% of the participants had worked for less than 5 years. The average daily working hours were 9.25 ± 1.25. More than 50% of the respondents reported short or long-time working hours. In addition, the participants who have not received mental health education have higher daily working hours (*p* < 0.05). The average sleeping time was 7.41 ± 1.07 h (Table 1).

**TABLE 1:** General characteristics information of the participants.

	Have you received mental health education?			
	
	Yes (*n* = 335)	No (*n* = 279)	Total (*N* = 614)	*P*-value
Age	27.42 ± 8.04	26.45 ± 7.15	26.98 ± 7.66	0.12
BMI	19.85 ± 2.21	19.97 ± 2.11	19.91 ± 2.16	0.50
**Gender**				
Male	35 (10.45%)	29 (10.39%)	64 (10.42%)	0.98
Female	300 (89.55%)	250 (89.61%)	550 (89.58%)	
**Educational level**				
Medium	24 (7.16%)	18 (6.45%)	42 (6.84%)	0.59
High	311 (92.84%)	261(93.55%)	564 (93.16%)	
**Marital status**				
Unmarried	206 (61.49%)	180 (64.52%)	386 (62.87%)	0.68
Married	124 (37.01%)	94 (33.69%)	218 (35.50%)	
Divorced	5 (1.49%)	5 (1.79%)	10 (1.63%)	
**Kindergarten attributes**				
Government-run kindergarten	154 (45.97%)	113 (40.50%)	267 (43.49%)	0.17
Private-run kindergarten	181 (54.03%)	166 (59.50%)	347 (56.51%)	
**Number of children**				
None	227 (67.76%)	196 (70.25%)	423 (68.89%)	0.79
One child	82 (24.48%)	62 (22.22%)	144 (23.45%)	
Two or more children	26 (7.76%)	21 (7.53%)	47 (7.65%)	
**Monthly income**				
< 3000yuan	143 (42.69%)	124 (44.44%)	267 (43.49%)	0.79
3000-4500yuan	118 (35.22%)	100 (35.84%)	218 (35.50%)	
4501-6000yuan	48 (14.33%)	39 (13.98%)	87 (14.17%)	
> 6000yuan	26 (7.76%)	16 (5.73%)	42 (6.84%)	
**Working experience**				
< 1 year	100 (29.85%)	105 (37.63%)	205 (33.39%)	0.14
1-5 years	123 (36.72%)	98 (35.13%)	221 (35.99%)	
5-10 years	53 (15.82%)	31 (11.11%)	84 (13.68%)	
More than 10 years	59 (17.61%)	45 (16.13%)	104 (16.94%)	
Daily working time	9.13 ± 1.28	9.39 ± 1.21	9.25 ± 1.25	0.013*
Sleeping Time	7.49 ± 1.14	7.32 ± 0.98	7.41 ± 1.07	0.058

Data are presented as mean ± SD for continuous measures, and n (%) for categorical measures; * *p*-value demonstrates the significance of results < 0.05, 95% CI = 95% confidence interval.

Table 2 summarizes the prevalence of symptoms among subscales of the SCL-90 between the respondents. Notably, 60% of participants showed that they might have mental health symptoms (GSI-T score ≥ 63). Nearly 1 in 10 (7%) preschool teachers suffered moderate to serious symptoms of obsessive-compulsive disorder, and 6.35% of participants suffered moderate to serious interpersonal sensitivity symptoms. Additionally, the positive detection rate of participants who have suffered moderate to serious symptoms of somatization, terror, diet, and sleep, depression, anxiety, hostility, paranoia, and psychotic are 6.19, 5.70, 4.89, 4.56, 4.56, 4.23, 4.23, and 1.79%, respectively (Table 2).

**TABLE 2:** Mental symptoms status diagnosed by SCL-90.

Subscales	No symptom	Mild symptom	Moderate to serious symptoms
Somatization	469 (76.38%)	107 (17.43%)	38 (6.19%)
Obsessive-compulsive disorder	432 (70.36%)	139 (22.64%)	43 (7.00%)
Interpersonal sensitivity	434 (70.68%)	141 (22.96%)	39 (6.35%)
Depression	498 (81.11%)	88 (14.33%)	28 (4.56%)
Anxiety	506 (82.41%)	80 (13.03%)	28 (4.56%)
Hostility	502 (81.76%)	86 (14.01%)	26 (4.23%)
Terror	448 (72.96%)	131 (21.34%)	35 (5.70%)
Paranoia	483 (78.66%)	105 (17.10%)	26 (4.23%)
Psychosis	505 (82.25%)	98 (15.96%)	11 (1.79%)
Appetite and sleep problems	57 (74.43%)	127 (20.68%)	30 (4.89%)
Psychological problems	245 (39.90%)		369 (60.1%)

All data are shown as %. Psychological problems (GSI-T-score > 63).

Table 3 displays the subscale score of SCL-90 among preschool teachers who received mental health education or not. The respondents who have not received mental health education reported higher mean scores of all the nine symptom dimensions of SCL-90 than those respondents who have received mental health education (*p* < 0.005).

**TABLE 3:** Scores of subscales of SCL-90.

Subscales of SCL-90	Total	Have you received mental health education?		
		
	*N* = 614	No(*n* = 279)	Yes(*n* = 335)	*P*-value
Somatization	1.64 ± 0.73	1.78 ± 0.79	1.52 ± 0.65	< 0.001
Obsessive-compulsive disorder	1.72 ± 0.70	1.84 ± 0.74	1.61 ± 0.65	< 0.001
Interpersonal sensitivity	1.71 ± 0.70	1.82 ± 0.76	1.62 ± 0.64	< 0.001
Depression	1.57 ± 0.66	1.66 ± 0.69	1.49 ± 0.62	< 0.001
Anxiety	1.55 ± 0.62	1.65 ± 0.68	1.46 ± 0.57	0.001
Hostility	1.50 ± 0.60	1.59 ± 0.63	1.43 ± 0.57	< 0.001
Phobic Anxiety	1.66 ± 0.68	1.78 ± 0.73	1.56 ± 0.61	0.001
Paranoia	1.57 ± 0.60	1.68 ± 0.66	1.49 ± 0.54	< 0.001
Psychosis	1.54 ± 0.52	1.61 ± 0.55	1.47 ± 0.50	< 0.001

All data are shown as the mean ± standard deviation. SCL-90, Symptom Checklist-90.

The multivariable-adjusted association between SCL-90 GSI-T score and mental health education with different work time is summarized in Table 4. In all models, SCL-90 scores were negatively associated with mental health education for all participants. Participants who had received mental health education had lower mean SCL-90 GSI-T scores. In model 1, participants who have not received mental health education had a higher GSI-T score (β = −1.40, 95% CI = −2.29, −0.52), and the associations remained significant after adjusting for sociodemographic and working background variables in models 2 and 3 (β_2_ = −1.38; β_3_ = −1.30). Additionally, a negative association was also found in the normal working hours group in different subgroup analyses of working hours (β = −1.74, 95% CI = −3.03, −0.45). However, in the long overtime hours group, although a greater correlation was found (-3.93, 95% CI = −9.99, 2.12), this difference was not significant (*p* > 0.05).

**TABLE 4:** The association between SCL-90 GSI-T score and mental health education by different overtime level.

GSI-T SCORE	All β(95%CI)	Normal β(95%CI)	Short overtime β(95%CI)	long-overtime β(95%CI)
**Model1**				
MH Education	−1.40*(−2.29, −0.52)	−1.66*(−2.87, −0.45)	−1.03(−2.11, 0.043)	−2.03(−6.95, 2.90)
**Model2**				
MH Education	−1.38*(−2.28, −0.48)	−1.56*(−2.80, −0.31)	−1.10*(−2.19, −0.006)	−2.93(−7.96, 2.09)
**Model3**				
MH Education	−1.30*(−2.21, −0.40)	−1.74*(−3.03, −0.45)	−1.07(−2.18, 0.05)	−3.93(−9.99, 2.12)

*P-value demonstrates the significance of results < 0.05, 95% CI = 95% Confidence Interval; Model 1: Adjusted age, BMI, gender, marital status, and the number of children. Covariates Model 2: As of model 1, mutually adjusted education level, job property, working experience, and monthly income. Covariates Model 3: As of model 2, mutually adjusted for sleeping time, eating behavior of breakfast, the frequency of moderated exercise, and history of allergy. MH, mental health. The reference group included participants who had not received mental health education.

## Discussion

Teaching is a stressful and challenging profession. However, it has been demonstrated that Chinese primary and secondary school teachers reported high psychological distress, especially in anxiety, sleep disturbance, and somatic symptoms during and after the COVID-19 pandemic (Lizhi et al., 2021; Wei et al., 2021). However, studies focused on mental health of preschool teachers are still few during and after the COVID-19 pandemic. This study aimed to evaluate the psychological problems and assess the association between mental health education and the SCL-90 scores among preschool teachers in southwest China. We found that preschool teachers probably need more mental health education due to a high rate of reported psychological problems and the negative association between mental health education and psychological problems.

In this study, more than 60% of preschool teachers reported psychological problems, which is higher than the previous research, which also used the SCL-90 scale in China before the COVID-19, which showed that 9–15% of the participants had obvious psychological problems (Li et al., 2004; Fan et al., 2016). A study conducted in southwestern China in February 2020 showed that the prevalence of anxiety and depression was high (Lei et al., 2020). The psychological problems of teachers increased during COVID-19, which were related to the reason that the closed situation accelerated the transformation of the teaching mode and decreased social activities (Aperribai et al., 2020). In contrast, the SCL-90 score of the population at the beginning of the pandemic was higher than the score in our study, which was surveyed at the end of 2020 (Bin-Yuan et al., 2020). We speculate that with the resumption of the epidemic, the order of lockdown and stay-at-home had been abolished, which resulted in a reduction in mental health problems.

The subscale score of SCL-90 showed that the mental health of participants in Southwest China is in trouble. The psychological problems of respondents are mainly manifested in obsessive-compulsive symptoms, interpersonal relationships, terror, and somatization symptoms, which are similar to the previous studies conducted among Chinese primary and secondary school teachers (Lizhi et al., 2021; Wei et al., 2021). Particularly, the prevalence of obsessive-compulsive disorder was highest. Preschool teachers always worry about the safety of preschoolers who are unable to take care of themselves, which might lead to the obsessive-compulsive disorder that has been demonstrated as a product of insecurity (Xuewu and Deqin, 2011). And the sense of extreme insecurity is prone to develop the preschool teachers’ pursuit of a perfect personality. Excessive self-discipline may also contribute to obsessive-compulsive disorder (Fei, 2009). Currently, preschool teachers are usually given higher social expectations while they had very low social status (Hsu et al., 2019). Thus, aspiration of approval might lead to personal compulsive tendencies. With a high rate of reported psychological problems, more education is probably needed for preschool teachers to relieve and prevent psychological problems.

Moreover, our study found that interpersonal sensitivity is common that is of concern, which is strongly associated with negative basic perceptions about the self and may mediate adults to abuse children (Otsuka et al., 2017; Mohammadian et al., 2018). Somatization is a tendency to experience and communicate physical pain in response to social and psychological stress and seek medical help for this (Lipowski, 1988). Previous studies have shown that while musculoskeletal disorders (MSDs) affect work efficiency and ability, it indirectly leads to psychological problems for preschool teachers (Otsuka et al., 2017). This is related to the heavy and complicated work that preschool teachers need to undertake. Studies have shown that proper physical activity can improve mental health by producing neurotransmitters and hormones (Lin and Kuo, 2013). However, this study found that preschool teachers in the Southwest have inadequate exercise, which may be related to the fact that they work long hours without enough time to exercise. More actions to improve the welfare of preschool teachers to achieve an effort-reward balance might be helpful.

Although the government of China has responded quickly and comprehensively, appropriate mental health policies were released (Mo, 2020; Qiu et al., 2020), which encouraged local governments, primary and secondary schools, and childcare institutions to carry out mental health education. However, only half of the preschool teachers reported receiving mental health education. This might be related to whether the local government or kindergarten provides mental health education, and unvalued mental health or the education on mental health promotion in Southwest China, including incomplete policies and insufficient promotion. Moreover, in China, most pieces of training are mainly short term, usually based on theory, lack long-term follow-up, and have close integration of theory and practice, which may lead to poor training effects (Gao et al., 2010). It is exceedingly necessary to carry out long-term follow-up research on mental health in the future.

In addition, we also found that 50% of preschool teachers reported having overtime work, which may result in not having enough time to participate in mental health education organized by local government or kindergartens. As work hours increase, it may lead to fatigue, stress, and sleep disturbance (Bannai et al., 2015). Previous studies indicated that overtime may increase the risk of depression (Watanabe et al., 2016). A similar finding of this study showed that high overtime levels harm mental health scores. Although 1 year has passed since the COVID-19 outbreak, the virus has continued to mutate over the year, with new episodes being reported. Preschool teachers may report high levels of psychological stress due to lots of work on the prevention and control of COVID-19. However, the respondents who are at a high level over time in the event of receiving mental health education showed better mental health. This suggests that mental health education might be very effective in improving the health of preschool teachers.

Based on these findings, we propose some suggestions to help improve mental health or prevent mental anxiety among early childhood teachers in the southwest. First, the local government should continue mental health education and expand its accessibility so that more preschool teachers have the opportunity to participate, and managers of kindergartens could make mental health education a daily practice to increase the mental health literacy of early childhood teachers. Second, preschool teachers are encouraged to work more efficiently, thereby reducing overtime and strengthening home-based cooperation. In addition, preschool teachers are also encouraged to adopt a healthy lifestyle and kindergarten administrators could organize more health promotion activities for teachers, e.g., exercise competitions.

This study explored the role of mental health education in the psychological problems of preschool teachers and found some valuable results, but there were also some limitations: First, due to the cross-sectional nature of our study, the temporal relationship of the cause and effect cannot be determined. Second, the selection of samples is limited to the Southwest region of China, where the economy is not developed well compared with the East or South regions. The results may not apply to other populations. Third, the working position of teachers was not considered (e.g., teaching academic year children), although the work experience of the kindergarten teachers was adjusted. Future research should consider the impact of working positions on kindergarten teachers. In addition, this study adopts the format of a questionnaire survey, which may lead to information and recall bias. Furthermore, evaluation of work stress and interpersonal relationships are not included in this study. It is recommended that more long-term tracking research is needed in the future to determine causal timing and ensure the effectiveness of psychological training; research objects can be expanded to different parts of China and multiple provinces.

## Conclusion

Few studies focus on the current status of mental health of preschool teachers in Southwest China. The results of the study demonstrated the significance of constructing the education of promoting preschool teachers’ mental health, and it could enlighten the government or managers of kindergartens to improve the psychological stress of preschool teachers through mental health education, especially for those with the pressure of overtime. It is recommended that local governments or work units should organize more mental health education for kindergarten teachers to improve their mental health as well as their teaching professionalism.

## Data availability statement

The raw data supporting the conclusions of this article will be made available by the authors, without undue reservation.

## Ethics statement

The studies involving human participants were reviewed and approved by Ethics Committee Review Committee of Chongqing Collaborative Innovation Center for Functional Food in Chongqing University of Education (202012HS02). The patients/participants provided their written informed consent to participate in this study.

## Author contributions

YY and TW conducted the statistical analyses of the data and prepared the manuscript. SW, YZ, and JG helped to revise the manuscript and data collection. JZ helped to review and provide critical comments to the manuscript. All authors checked and proofread the final version of the manuscript.
